# Development and validation of a prediction model for early identification of critically ill elderly COVID-19 patients

**DOI:** 10.18632/aging.103716

**Published:** 2020-10-06

**Authors:** Jue Liu, Liyuan Tao, Zhancheng Gao, Rongmeng Jiang, Min Liu

**Affiliations:** 1 Department of Epidemiology and Biostatistics, School of Public Health, Peking University, Beijing, China; 2 Research Center of Clinical Epidemiology, Peking University Third Hospital, Beijing, China; 3 Department of Respiratory and Critical Care Medicine, Peking University People's Hospital, Beijing, China; 4 Centre for Infectious Disease, Beijing Ditan Hospital, Capital Medical University, Beijing, China

**Keywords:** coronavirus disease, COVID-19, elderly patients, prediction model, critically ill

## Abstract

In this study, we established a simple and practical tool for early identification of potentially high-risk individuals among elderly COVID-19 patients. Included were 2106 laboratory-confirmed COVID-19 patients aged 60 years and above in 30 provinces of mainland China. Using discrimination (the area under the receiver-operator characteristic curve [AUC]) and calibration (Hosmer-Lemeshow goodness-of-fit test and calibration plots), a nomogram for predicting critically ill cases was developed, and its performance was examined using an internal validation cohort (444 patients) and external cohort (770 patients). The proportion of critically ill patients was 11.8% (248/2106). The most common symptoms at the onset of illness were fever (66.6%), cough (34.1%), fatigue (23.3%), and expectoration (23.6%). Older age, history of chronic obstructive pulmonary disease, fever, fatigue, shortness of breath, and lymphocyte percentage lower than 20% at admission were associated with increased risk of becoming critically ill. The AUCs for the six-variable-based nomogram were 0.77 (95% CI: 0.73-0.82), 0.73 (95% CI: 0.67-0.79), and 0.77 (95% CI: 0.71-0.83) in the development, internal validation, and external validation cohorts, respectively. This six-variable-based nomogram could potentially serve as a practical and reliable tool for early identification of elderly COVID-19 patients at high risk of becoming critically ill.

## INTRODUCTION

In December 2019, several pneumonia cases of unknown origin were reported in Wuhan, Hubei province of China. A novel coronavirus (severe acute respiratory syndrome coronavirus 2, SARS-CoV-2) was isolated by Chinese scientists [1]. Since January 2020, the coronavirus disease 2019 (COVID-19) has spread rapidly to all 31 China provinces, as well as around the world. The World Health Organization (WHO) has declared the outbreak of COVID-19 as a public health emergency of international concern. As of June 18, 2020, a total of 8,242,999 confirmed COVID-19 cases and 445,535 deaths (representing 5.4% mortality) have been reported globally [2].

Among all COVID-19 patients, the elderly population is especially at high risk of severe illness and death [3–7]. A national epidemiology study in China reported that 31.2% of 44,672 confirmed COVID-19 cases were patients aged 60 years and above, and 81% of all deaths were patients aged 60 years and above [5]. Case fatality rate was high in critically ill cases (49.0%) and in elderly patients (14.8% for ≥80 years and 8.0% for 70-79 years), compared with case fatality rate (2.3%) of all 44,672 confirmed cases in China [5]. A recent study showed that increased risk of in-hospital death was associated with older age (odds ratio 1.10, 95% CI 1.03–1.17) among 191 adult inpatients in Wuhan [4]. A retrospective cohort study of 201 patients showed that older age (≥65 years) was associated with greater risk of development of ARDS (hazard ratio 3.26, 95% CI 2.08-5.11) and death (hazard ratio 6.17, 95% CI 3.26-11.67) [6]. Arentz and colleagues reported that the mean age of critically ill patients with COVID-19 was 70 years (range 43-92 years) and mortality was 67% in 21 patients in a single center in Washington State [8]. Given the rapid spread of COVID-19 worldwide, an early identification of potentially critical patients is crucial to reduce the case fatality rate, especially for elderly patients.

A recent single-centered retrospective observational study of 52 critical COVID-19 patients reported that non-survivors were older (median age 64.6 years vs 51.9 years), more likely to develop ARDS (81% vs 45%), and more likely to receive mechanical ventilation (94% vs 35%), compared with survivors [3]. However, the risk factors for critically ill elderly patients remain unknown, and information on early identification of potentially critical patients in elderly population is scarce.

In this study, we aimed to establish and validate a simple and practical tool for early identification of high-risk critically ill cases among elderly COVID-19 patients in mainland China. Using demographics, medical history, onset symptoms, and simple physical examination at admission, we generated a convenient nomogram model for early identification of elderly COVID-19 patients who are at a high risk of becoming critically ill.

## RESULTS

**Figure 1:**
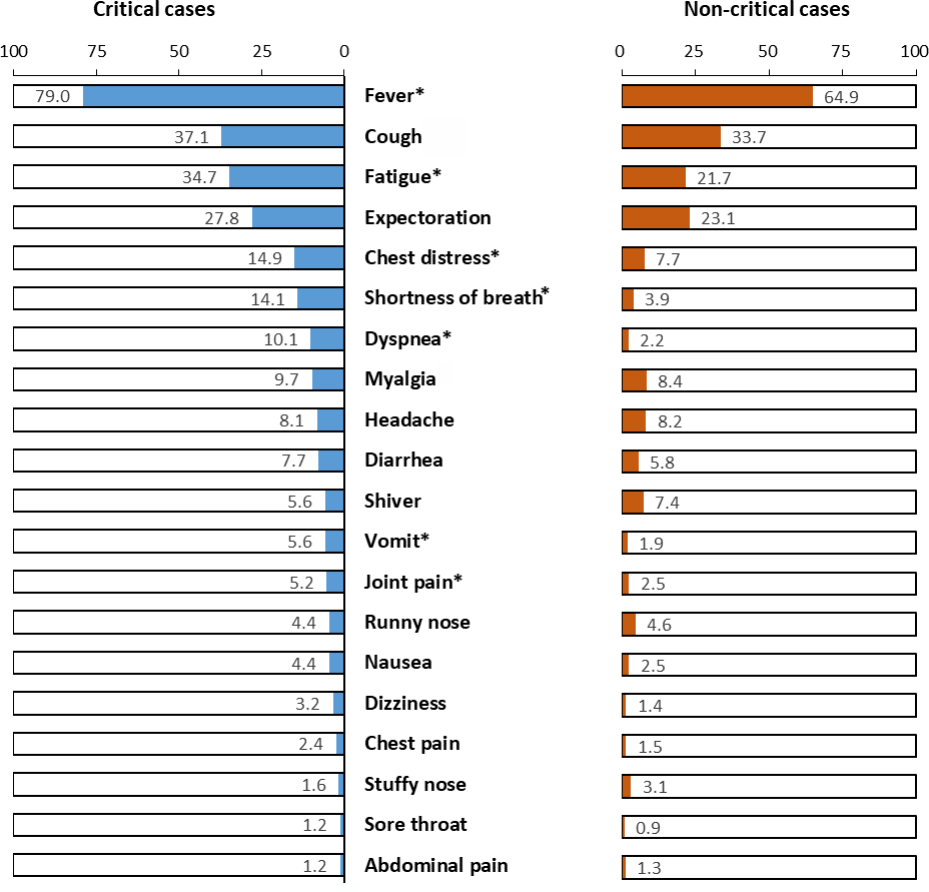
**Symptoms at onset of illness in critical and non-critical cases (n=2106).** Notes, *P<0.05.

In total, 2106 laboratory-confirmed COVID-19 patients aged 60 years and above were included in this study. The median age of the patients was 67 years (IQR: 63, 73). 52.3% (1102/2106) of patients had an underlying medical condition. 11.8% (248/2106) of the elderly COVID-19 patients became critically ill. The most common symptoms at onset of illness were fever (66.6%), cough (34.1%), fatigue (23.3%), and expectoration (23.6%). Compared with non-critical cases, critically ill cases had higher proportion of fever (79.0% vs 64.9%), fatigue (34.7% vs 21.7%), chest distress (14.9% vs 7.7%), shortness of breath (14.1% vs 3.9%), dyspnea (10.1% vs 2.2%), vomiting (5.6% vs 1.9%), and joint pain (5.2% vs 2.5%) (all p<0.05, Figure 1). With the progress of China's comprehensive prevention and control measures, the median days from onset of symptoms to COVID-19 diagnosis were significantly reduced, from 9-10 days before 23 January 2020 to 3-4 days after 1 February 2020 (Supplementary Figure 1, p<0.001).

By the date of disease onset, the patients were divided into development, internal validation, and external validation cohorts. Characteristics of the COVID-19 critical patients in the development cohort (n=892), internal validation cohort (n=444), and external validation cohort (n=770) were similar (Supplementary Table 2).

In the development cohort, the proportion of critically ill cases was 13.2% (118/774); the percentage of critically ill patients was significantly higher in older patients, males, patients living in rural areas, patients with comorbidities (hypertension, diabetes, chronic obstructive pulmonary disease, or chronic kidney disease), high body temperature, high white blood cell count, low lymphocyte percentage, low lymphocyte count, and high neutrophil percentage (all p<0.05, Supplementary Table 2). In the multivariable logistic regression model, older age (adjusted odds ratio [aOR] 2.73 for 70-79 years, 95% CI: 1.74-4.29; aOR=3.78 for 80 years and above, 95% CI: 1.96-7.28), chronic obstructive pulmonary disease (aOR 2.33, 95% CI: 1.16-4.67), fever (aOR 1.97 for temperature 38.1-39°C, 95% CI: 1.11-3.48; aOR 5.05 for temperature 39°C and above, 95% CI: 1.90-13.44), fatigue (aOR 1.63, 95% CI: 1.06-2.53), shortness of breath (aOR 2.74, 95% CI: 1.36-5.52), and lymphocyte percentage < 20% (aOR 2.28, 95% CI: 1.48-3.53) were associated with a significantly increased risk of critical illness (p<0.05, Table 1). In sensitivity analysis, the robustness of the model was established by including age as a continuous variable (Supplementary Table 1), instead of a categorical variable.

**Table 1:** Risk factors for critical illness among COVID-19 patients in the development cohort: univariable and multivariable models.

	**cOR (95% CI)**	**p**		**aOR (95% CI)**	**p**
Age					
60-69	1.00			1.00	
70-79	2.82 (1.84-4.32)	<0.001		2.73 (1.74-4.29)	<0.001
≥80	3.55 (1.94-6.50)	<0.001		3.78 (1.96-7.28)	<0.001
Chronic obstructive pulmonary disease					
No	1.00			1.00	
Yes	3.31 (1.77-6.20)	<0.001		2.33 (1.16-4.67)	0.017
Temperature (°C)					
<37.3	1.00			1.00	
37.3-38	1.08 (0.62-1.86)	0.785		1.25 (0.70-2.22)	0.455
38.1-39	1.88 (1.10-3.21)	0.020		1.97 (1.11-3.48)	0.020
>39	5.05 (2.08-12.23)	<0.001		5.05 (1.90-13.44)	0.001
Fatigue					
No	1.00			1.00	
Yes	1.98 (1.33-2.96)	0.001		1.63 (1.06-2.53)	0.027
Shortness of breath					
No	1.00			1.00	
Yes	4.31 (2.33-8.00)	<0.001		2.74 (1.36-5.52)	0.005
Lymphocyte percentage (%)					
20-40	1.00			1.00	
<20	2.79 (1.85-4.21)	<0.001		2.28 (1.48-3.53)	<0.001
>40	1.21 (0.49-2.97)	0.684		1.54 (0.60-3.93)	0.370

cOR, crude odds ratio; aOR, adjusted odds ratio.

In the multivariable model, the significant variables were selected using a backward procedure from their age group, sex, region, history of disease (hypertension, diabetes, coronary heart disease, chronic obstructive pulmonary disease, chronic kidney disease, or chronic liver disease), body temperature (<37.3°C, 37.3-38°C, 38.1-39°C, or >39°C), days from onset to diagnosis, white blood cell count, lymphocyte percentage, lymphocyte count, neutrophil percentage, symptoms (cough, fatigue, expectoration, chest distress, myalgia, shiver, headache, shortness of breath, dyspnea, diarrhea, runny nose, nausea, joint pain, vomit, stuffy nose, chest pain, dizziness, abdominal pain, or sore throat).

**Figure 2:**
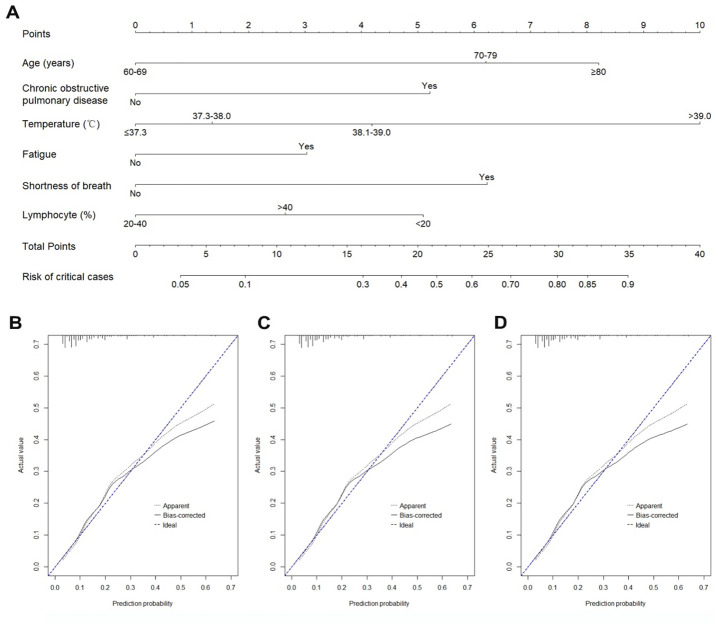
**Nomogram and calibration curves for predicting the risk of critically illness among COVID-19 patients.** (**A**) Nomogram model for predicting the risk of critically ill cases among COVID-19 patients. (**B**) Calibration curves for the nomogram in the development cohort. (**C**) Calibration curves for the nomogram in the internal validation cohort. (**D**) Calibration curves for the nomogram in the external validation cohort.

We established a nomogram based on the model selected variables (age, chronic obstructive pulmonary disease, body temperature, fatigue, shortness of breath, and lymphocyte percentage) to predict critical illness (Figure 2). In the development cohort, the AUC for the prediction nomogram was 0.77 (95% CI: 0.73-0.82). In the internal validation cohort, the proportion of critically ill cases was 13.5% (60/444). The AUC (0.73, 95% CI: 0.67-0.79), the Hosmer-Lemeshow goodness-of-fit test (p=0.991), and the calibration curve showed good discrimination and calibration of nomogram in the internal validation cohort (Figure 2).

**Figure 3:**
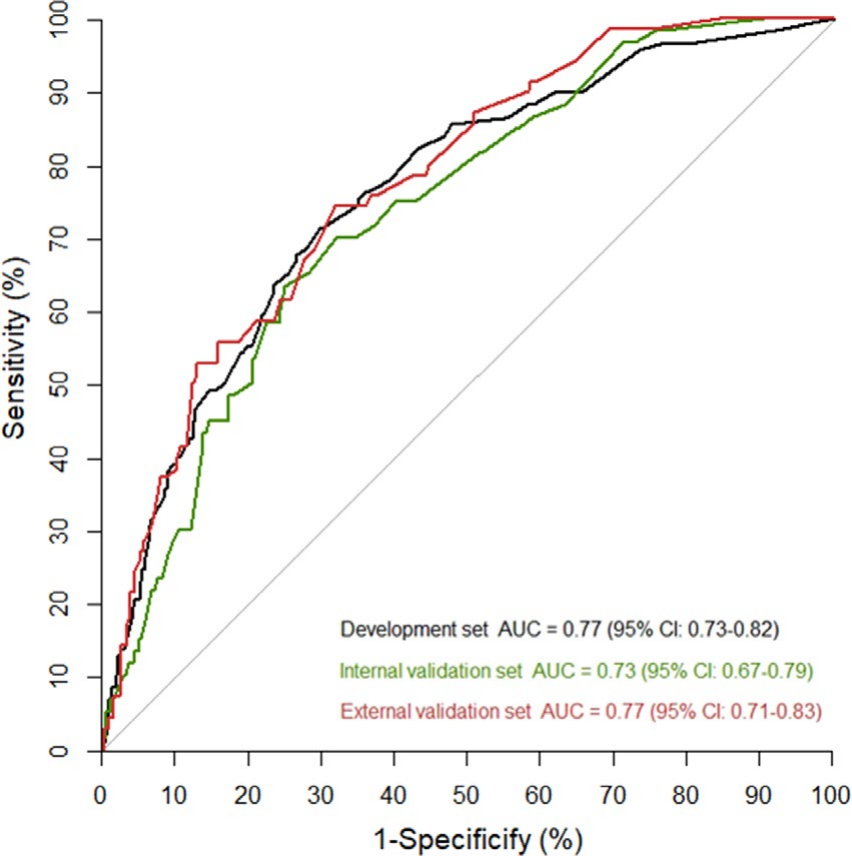
**Areas under the curve (AUC) in the nomogram model for predicting the risk of critically ill cases among COVID-19 patients in the development and validation cohorts.**

In the external validation cohort, the proportion of critical illness was 9.1%, which was significantly lower than in the development (13.2%) and internal validation (13.5%) sets (p=0.015). Prediction accuracy of the nomogram was stable in the external validation cohort with the AUC of 0.77 (5% CI: 0.71-0.83). The Hosmer-Lemeshow test (p=0.393) and calibration curve (Figure 2D) also showed a good calibration. AUCs of nomogram in the development and validation cohorts were similar (Figure 3).

## DISCUSSION

To our knowledge, this is the first study on development and validation of a simple nomogram for early prediction of critically ill cases among elderly COVID-19 patients. Elderly patients are at a higher risk of becoming critically ill and die from COVID-19 infection compared to other age groups [3]. Thus, an early detection of potentially critical cases in elderly patients is essential for timely intervention to prevent death. We found that the nomogram prediction model in our study showed a good accuracy, with AUC above 0.7 in the internal and external validation cohorts. The nomogram prediction model consisted of six simple variables (age, history of chronic obstructive pulmonary disease, body temperature, feeling fatigue, shortness of breath, and lymphocyte percentage), which are easily accessible in the clinical practice and in the community. Our data suggested that this simple nomogram could be a useful and practical tool for early screening of a high-risk population of critically ill cases among elderly COVID-19 patients, especially in countries with relatively limited medical resources.

As for the risk factors of COVID-19 critically ill cases among the elderly, we found that older age (aOR 2.73-3.78), chronic obstructive pulmonary disease (aOR 2.33), high body temperature (aOR 1.97-5.05), fatigue (aOR 1.63), shortness of breath (aOR 2.74), and lymphocyte percentage less than 20% (aOR 2.28) were associated with critical illness in the multivariable logistic regression model. A poor prognosis in older patients was reported in previous studies [3, 5, 7, 9]. The association between chronic obstructive pulmonary disease and critical illness might be caused by the compromised respiratory status on admission (the primary driver of disease severity). Lymphocytopenia in COVID-19 patients was also reported in previous studies; the median lymphocyte count was lower in severe cases than in non-severe cases, and was lower in non-survivors than in survivors [3, 10]. Severe cases tend to have lower lymphocytes counts, higher neutrophil to lymphocyte ratios, as well as lower percentages of monocytes, memory helper T cells, and basophils [11].

Our data showed that the proportion of critically ill cases was 11.8% among elderly COVID-19 patients in 760 hospitals outside of Hubei; this proportion was lower in later period (9.1%) than in early period (13.2-13.5%). This might be related to the improvements in diagnosis and treatment in later period of the COVID-19 outbreak. Early diagnosis and treatment of COVID-19 patients are important to prevent the critical illness. We found that the number of days from onset to COVID-19 diagnosis was reduced significantly in both non-critical and critical cases, from 9-10 days before 23 January 2020, to 3-4 days after 1 February 2020. The reduced time from onset to diagnosis might have also contributed to the decreased number of critically ill cases.

However, it is worth noting that the proportion of critically ill cases in elderly population in our study (11.8%) was much higher than reported in the general population (4.7%). There are no previous studies reporting the total proportion of critically ill cases among elderly COVID-19 patients in mainland China outside of Hubei. Xu and colleagues reported that the COVID-19 severity of 62 patients with median age of 41 (IQR 32-52) in Zhejiang province was relatively mild, with only 2 patients aged above 65 [12]. In the largest national epidemiology study, the proportion of critically ill cases was found 4.7% among 44627 confirmed COVID-19 patients of all age groups (using all cases reported through February 11, 2020), without reporting the proportion of critically ill cases among different age groups [5]. Yang and colleagues observed that non-survivors (64.6 years) were older compared to survivors (51.9 years) among 52 patients in Wuhan [3]. A retrospective cohort of 487 patients reported that patients with severe symptoms (56±17 years) were older compared to mild cases (45±19 years) [13]. Wu and colleagues reported that an older age (≥65 years) was associated with a greater risk of death (HR=6.17, 95% CI=3.26-11.67) [6]. Based on previous studies [3, 5, 6, 14], evidence suggests that elderly population is the most susceptible to COVID-19 related death, and should thus receive more attention.

Consistent with previous studies [3, 4, 10, 15], we found that fever and cough were the most common symptoms at the onset of illness. Moreover, we found that critically ill cases had a higher occurrence of fever (79.0% vs 64.9%), fatigue (34.7% vs 21.7%), shortness of breath (14.1% vs 3.9%), chest distress (14.9% vs 7.7%), dyspnea (10.1% vs 2.2%), vomiting (5.6% vs 1.9%) and joint pain (5.2% vs 2.5%) compared with non-critical cases. The different proportion of symptoms at the onset of illness in critical and non-critical cases might be related to the severity of disease, comorbid conditions, and affected organs. A previous study compared the symptoms between 20 survivors and 32 non-survivors in a single-centered, retrospective, observational study, and showed that the proportion of fatigue (or malaise) was higher in non-survivors (44%) than in survivors (20%), although not statistically significant [3]. Another study compared 109 dead patients and 116 recovered patients in Wuhan, and showed that the patients who died had a higher proportion of fever before admission (87.2% vs 81.0%), fatigue (27.5% vs 23.3%), dyspnea (70.6% vs 19.0%), and expectoration (32.1% vs 12.1%), although only dyspnea and expectoration were statistically significant [9]. Guan and colleagues reported that the proportion of shortness of breath was higher in severe cases (37.6%) than in non-severe cases (15.1%) during hospitalization [10]. Further studies are needed to identify the underlying mechanisms of COVID-19 pathogenesis in critically ill cases.

This study has several limitations. First, we did not include elderly COVID-19 patients in Hubei province because of lacking data. However, we included elderly patients in 760 hospitals outside of Hubei province in mainland China; this was the first and largest sample of this kind. Second, because specific information on treatment and recovery, such as the date of discharge, and the date of progression to critically illness, was not collected, we could not use time-to-event analysis in this study. However, the aim of this study was to establish a simple tool for an early detection of patients who are at a high-risk for developing critical COVID-19 symptoms. Third, the validation was only conducted in mainland China in this study. Further external validation studies of the nomogram model outside of China are needed. Fourth, patients who received ICU care and mechanical ventilation were selected by local clinicians; thus, this might have caused a bias because of a lack of standard definitions.

Nearly 10% of COVID-19 patients were critically ill in the elderly population. Using only six easily accessible variables, a simple nomogram detected critically ill cases in elderly COVID-19 patients with an AUC of 0.77 (95% CI: 0.73-0.82), 0.73 (95% CI: 0.67-0.79) and 0.77 (95% CI: 0.71-0.83) in the development, internal validation, and external validation cohorts, respectively. This model may serve as a simple and reliable predictive tool for an early identification of elderly COVID-19 patients who are at a high risk of becoming critically ill, especially in countries with relatively limited medical resources.

## MATERIALS AND METHODS

### Study design and participants

The study was supported by National Health Commission of China. From 760 hospitals of 30 provinces in mainland China, the data of 2106 laboratory-confirmed COVID-19 patients aged 60 years and above, whose date of diagnosis was before February 25, 2020, were collected. The final date of follow-up was March 8, 2020. The data were divided into development cohort, internal validation cohort, and external validation cohort by the date of disease onset. 1336 patients whose date of onset was before February 2, 2020, were assigned into the development and internal validation cohorts. Computer-generated random numbers were used to assign these subjects into the development cohort (consisting of 892 samples) and the internal validation cohort (consisting of 444 samples). 770 COVID-19 patients, whose date of onset was after February 2, 2020, were assigned into the external independent validation cohort. Data collection and analysis of cases were determined by the National Health Commission of China to be part of a continuing public health response to control the outbreak, and were thus considered exempt from institutional review board approval.

### Procedures

The diagnosis of COVID-19 was conducted by local healthcare workers according to the national diagnosis and treatment protocol for COVID-19 released by the National Health Commission [9]. A confirmed COVID-19 case was defined as a positive result on high-throughput sequencing or real-time reverse-transcriptase-polymerase-chain-reaction (RT-PCR) assay of nasal and pharyngeal swab specimens [16, 17]. Brief medical records reported to the National Health Commission were used for analysis of the demographics (age, sex, occupation, and region), medical history (hypertension, diabetes, coronary heart disease, chronic obstructive pulmonary disease, chronic kidney disease, chronic liver disease, etc), onset of symptoms (fever, cough, fatigue, expectoration, chest distress, shortness of breath, dyspnea, diarrhea, etc.), date of onset, date of diagnosis, and physical examination at admission (body temperature, white blood cell count, lymphocyte percentage, lymphocyte count, neutrophil percentage).

### Outcomes

The primary outcome was a critical illness. Critically ill cases were defined as cases meeting any of the following criteria: 1) respiratory failure and requiring mechanical ventilation; 2) shock; or 3) other organ failure that requires ICU care [16]. Diagnosis of critically ill cases was conducted by local healthcare workers according to the national diagnosis and treatment protocol for COVID-19 released by the National Health Commission [16]. Non-critical cases were composed of mild cases, moderate cases, and severe cases. Mild cases were defined as patients whose clinical symptoms were mild and there was no sign of pneumonia on imaging. Moderate cases were defined as patients showing fever and respiratory symptoms with radiological findings of pneumonia. Severe cases were defined as patients meeting any of the following criteria: 1) respiratory distress (≥30 breaths/ min); 2) oxygen saturation≤93% at rest; 3) arterial partial pressure of oxygen (PaO2)/ fraction of inspired oxygen (FiO2) ≤ 300mmHg (l mmHg=0.133kPa) [16]. The percentage of critically ill cases was defined as the ratio of critically ill cases to all laboratory-confirmed COVID-19 patients aged 60 and above.

### Statistical analysis

For age analysis, medians and interquartile range (IQR) were calculated. We used proportions to analyze the baseline characteristics of patients, including sex, occupation, region, and medical history. We used Chi-square test or Fisher's exact test to compare the proportions of COVID-19 critically ill cases of different characteristics in the development, internal, and external validation cohorts for categorical variables.

We developed a nomogram for prediction of critically ill cases using a multivariable logistic regression model. In the multivariable model, we analyzed potential risk factors related to critically ill cases, including age group (60-69 years, 70-79 years, or 80 years and above), sex (female or male), occupation (factory worker, farmer, retiree, house worker, business services staff, manager, or others), region (urban or rural), medical history (hypertension, diabetes, coronary heart disease, chronic obstructive pulmonary disease, chronic kidney disease, or chronic liver disease), body temperature (<37.3°C, 37.3-38°C, 38.1-39°C, or >39°C), days from onset to diagnosis (≤3 days, 4-7 days, or >7days), white blood cell count (<4×10^9^/L, 4-10×10^9^/L, or >10×10^9^/L), lymphocyte percentage (20%-40%, <20%, >40%), lymphocyte count (<1×10^9^/L, or ≥1×10^9^/L), neutrophil percentage (50-70%, <50%, or >70%), and symptoms (cough, fatigue, expectoration, chest distress, myalgia, shiver, headache, shortness of breath, dyspnea, diarrhea, runny nose, nausea, joint pain, vomit, stuffy nose, chest pain, dizziness, abdominal pain, or sore throat). We applied a backward procedure for variables selection for the multivariable logistic regression model. Regression coefficients were used to generate a nomogram. To examine robustness of the model, sensitivity analysis was conducted by including age as a continuous variable, instead of categorical variable in the model.

Nomogram model performance was assessed by examining discrimination and calibration in the development and validation cohorts. The discrimination was assessed by the area under the receiver-operator characteristic (ROC) curve (AUC) and its 95% CI. The calibration was constructed to examine the agreement between the predicted probabilities with the observed outcome, which was assessed by the Hosmer-Lemeshow goodness-of-fit test and calibration plots. The calibration plot was calculated by the 1000 repetitions Bootstrap resampling. Development and reporting of the prediction model followed the Transparent Reporting of a multivariable prediction model for Individual Prognosis Or Diagnosis (TRIPOD) Statement [18–20].

Statistical tests were done with R software (version 3.6.0) and SPSS (version 25.0). Statistical significance was set at two-sided p values less than 0.05.

## Supplementary Material

Supplementary Figure 1

Supplementary Table 1

Supplementary Table 2
