# Supplementary material for: Development and validation of a prediction model for early identification of critically ill elderly COVID-19 patients
Source: Aging (Albany NY). 2020 Oct 6;12(19):18822–32. doi: 10.18632/aging.103716 (PMC7732309; doi:10.18632/aging.103716)
Supplement: Supplementary Figure 1 [file aging-12-103716-s001..pdf]

SUPPLEMENTARY FIGURE

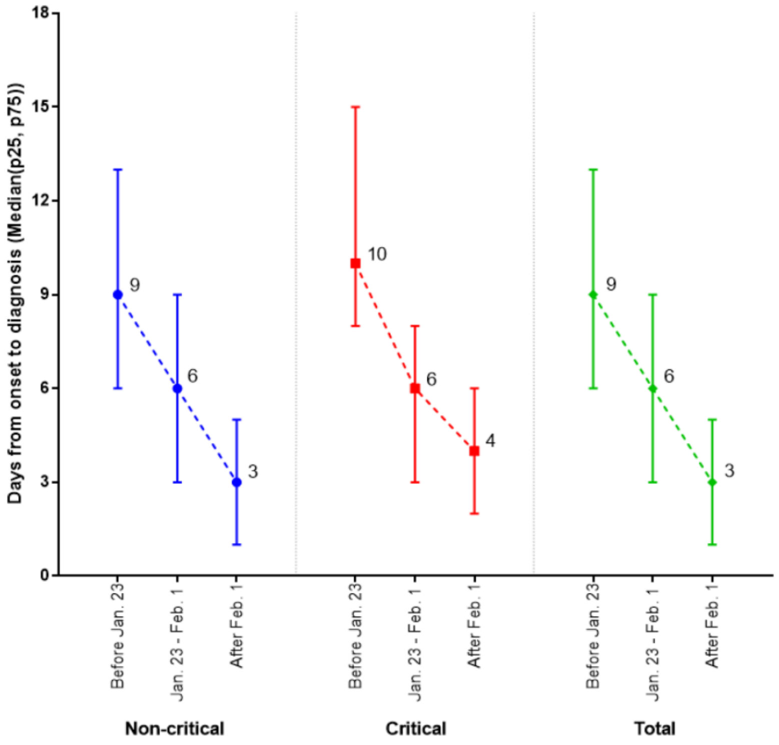

Supplementary Figure 1. Days from onset to diagnosis during different periods in critical and non-critical cases (n=2106).
