# Supplementary material for: Development and validation of a prediction model for early identification of critically ill elderly COVID-19 patients
Source: Aging (Albany NY). 2020 Oct 6;12(19):18822–32. doi: 10.18632/aging.103716 (PMC7732309; doi:10.18632/aging.103716)
Supplement: Supplementary Table 1 [file aging-12-103716-s002..pdf]

SUPPLEMENTARY TABLE

Supplementary Table 1. Sensitive analysis of the risk factors for critical illness among COVID-19 patients in the development cohort.

	cOR (95% CI)	p	aOR (95% CI)	p
Age	1.08 (1.06-1.11)	<0.001	1.09 (1.06-1.12)	<0.001
Chronic obstructive pulmonary disease				
No	1.00		1.00	
Yes	3.31 (1.77-6.20)	<0.001	2.20 (1.08-4.45)	0.029
Temperature (°C)				
<37.3	1.00		1.00	
37.3-38	1.08 (0.62-1.86)	0.785	1.28 (0.74-2.29)	0.407
38.1-39	1.88 (1.10-3.21)	0.020	2.11 (1.19-3.75)	0.011
>39	5.05 (2.08-12.23)	<0.001	5.05 (1.90-13.61)	0.001
Fatigue				
No	1.00		1.00	
Yes	1.98 (1.33-2.96)	0.001	1.69 (1.10-2.62)	0.018
Shortness of breath				
No	1.00		1.00	
Yes	4.31 (2.33-8.00)	<0.001	2.83 (1.41-5.71)	0.004
Lymphocyte percentage (%)				
20-40	1.00		1.00	
<20	2.79 (1.85-4.21)	<0.001	2.29 (1.47-3.55)	<0.001
>40	1.21 (0.49-2.97)	0.684	1.67 (0.65-4.28)	0.288

cOR, crude odds ratio; aOR, adjusted odds ratio.

In the multivariable model, the significant variables were selected using a backward procedure from age (as a continuous variable), sex, region, history of disease (hypertension, diabetes, coronary heart disease, chronic obstructive pulmonary disease, chronic kidney disease, or chronic liver disease), body temperature (<37.3°C, 37.3-38°C, 38.1-39°C, or >39°C), days from onset to diagnosis, white blood cell count, lymphocyte percentage, lymphocyte count, neutrophil percentage, symptoms (cough, fatigue, expectoration, chest distress, myalgia, shiver, headache, shortness of breath, dyspnea, diarrhea, runny nose, nausea, joint pain, vomit, stuffy nose, chest pain, dizziness, abdominal pain, or sore throat).
