# Supplementary material for: Development and validation of a prediction model for early identification of critically ill elderly COVID-19 patients
Source: Aging (Albany NY). 2020 Oct 6;12(19):18822–32. doi: 10.18632/aging.103716 (PMC7732309; doi:10.18632/aging.103716)
Supplement: Supplementary Table 2 [file aging-12-103716-s003..docx]

| **Supplementary Table 2. Characteristics of COVID-19 patients in the development, internal, and external validation cohorts** | | | | | | | | | | | | | | | | | |  |
| --- | --- | --- | --- | --- | --- | --- | --- | --- | --- | --- | --- | --- | --- | --- | --- | --- | --- | --- |
| **Characteristics** | **N** | **Development cohort (n=892)** | | |  | **Internal validation cohort (n=444)** | | |  | | **External validation cohort (n=770)** | | |  | **Total (n=2106)** | | |  |
|  |  | **Non-critical** | **Critical** | **p** |  | **Non-critical** | **Critical** | **p** |  | | **Non-critical** | **Critical** | **p** |  | **Non-critical** | **Critical** | **p** |  |
| **Demographics** |  |  |  |  |  |  |  |  |  | |  |  |  |  |  |  |  |  |
| Age (years) |  |  |  | <0.001^*^ |  |  |  | <0.001^*^ |  | |  |  | <0.001^*^ |  |  |  | <0.001^*^ |  |
| 60-69 | 1349 | 543 (91.3) | 52 (8.7) |  |  | 288(91.1) | 28(8.9) |  |  | | 416(95.0) | 22(5.0) |  |  | 1247(92.4) | 102(7.6) |  |  |
| 70-79 | 552 | 178 (78.8) | 48 (21.2) |  |  | 79(78.2) | 22(21.8) |  |  | | 201(89.3) | 24(10.7) |  |  | 458(83.0) | 94(17.0) |  |  |
| ≥80 | 205 | 53 (74.6) | 18 (25.4) |  |  | 17(63.0) | 10(37.0) |  |  | | 83(77.6) | 24(22.4) |  |  | 153(74.6) | 52(25.4) |  |  |
| Sex |  |  |  | 0.010^*^ |  |  |  | 0.118 |  | |  |  | 0.538 |  |  |  | 0.004^*^ |  |
| Female | 1096 | 413 (89.6) | 48 (10.4) |  |  | 208(88.9) | 26(11.1) |  |  | | 367(91.5) | 34(8.5) |  |  | 988(90.1) | 108(9.9) |  |  |
| Male | 1010 | 361 (83.8) | 70 (16.2) |  |  | 176(83.8) | 34(16.2) |  |  | | 333(90.2) | 36(9.8) |  |  | 870(86.1) | 140(13.9) |  |  |
| Occupation |  |  |  | 0.705^a^ |  |  |  | 0.634^a^ |  | |  |  | 0.798^a^ |  |  |  | 0.139^a^ |  |
| Factory worker | 20 | 10 (100.0) | 0 (0.0) |  |  | 3(75.0) | 1(25.0) |  |  | | 6(100.0) | 0(0.0) |  |  | 19(95.0) | 1(5.0) |  |  |
| Farmer | 666 | 212 (89.5) | 25 (10.5) |  |  | 114(89.1) | 14(10.9) |  |  | | 278(92.4) | 23(7.6) |  |  | 604(90.7) | 62(9.3) |  |  |
| Retiree | 858 | 346 (85.2) | 60 (14.8) |  |  | 150(84.3) | 28(15.7) |  |  | | 243(88.7) | 31(11.3) |  |  | 739(86.1) | 119(13.9) |  |  |
| House worker | 417 | 140 (85.9) | 23 (14.1) |  |  | 86(88.7) | 11(11.3) |  |  | | 143(91.1) | 14(8.9) |  |  | 369(88.5) | 48(11.5) |  |  |
| Business services staff | 55 | 26 (86.7) | 4 (13.3) |  |  | 16(84.2) | 3(15.8) |  |  | | 6(100.0) | 0(0.0) |  |  | 48(87.3) | 7(12.7) |  |  |
| Manager | 10 | 3 (100.0) | 0 (0.0) |  |  | 3(100.0) | 0(0.0) |  |  | | 4(100.0) | 0(0.0) |  |  | 10(100.0) | 0(0.0) |  |  |
| Others | 80 | 37 (86.0) | 6 (14.0) |  |  | 12(80.0) | 3(20.0) |  |  | | 20(90.9) | 2(9.1) |  |  | 69(86.3) | 11(13.8) |  |  |
| Region |  |  |  | 0.044^*^ |  |  |  | 0.358^*^ |  | |  |  | 0.744 |  |  |  | 0.190 |  |
| Urban | 1599 | 626 (87.9) | 86 (12.1) |  |  | 296(87.3) | 43(12.7) |  |  | | 497(90.7) | 51(9.3) |  |  | 1419(88.7) | 180(11.3) |  |  |
| Rural | 507 | 148 (82.2) | 32 (17.8) |  |  | 88(83.8) | 17(16.2) |  |  | | 203(91.4) | 19(8.6) |  |  | 439(86.6) | 68(13.4) |  |  |
| **History of disease** |  |  |  |  |  |  |  |  |  | |  |  |  |  |  |  |  |  |
| History of disease |  |  |  | <0.001^*^ |  |  |  | 0.037^*^ |  | |  |  | <0.001^*^ |  |  |  | <0.001^*^ |  |
| No | 1004 | 396 (92.1) | 34 (7.9) |  |  | 190 (90.0) | 21 (10.0) |  |  | | 345 (95.0) | 18 (5.0) |  |  | 931 (92.7) | 73 (7.3) |  |  |
| Yes | 1102 | 378 (81.8) | 84 (18.2) |  |  | 194 (83.3) | 39 (16.7) |  |  | | 355 (87.2) | 52 (12.8) |  |  | 927 (84.1) | 175 (15.9) |  |  |
| Hypertension |  |  |  | 0.007^*^ |  |  |  | 0.130 |  | |  |  | 0.002^*^ |  |  |  | <0.001^*^ |  |
| No | 1479 | 566 (88.7) | 72 (11.3) |  |  | 262 (88.2) | 35 (11.8) |  |  | | 506 (93.0) | 38 (7.0) |  |  | 1334 (90.2) | 145 (9.8) |  |  |
| Yes | 627 | 208 (81.9) | 46 (18.1) |  |  | 122 (83.0) | 25 (17.0) |  |  | | 194 (85.8) | 32 (14.2) |  |  | 524 (83.6) | 103 (16.4) |  |  |
| Diabetes |  |  |  | 0.024^*^ |  |  |  | 0.019^*^ |  | |  |  | <0.001^*^ |  |  |  | <0.001^*^ |  |
| No | 1844 | 676 (87.8) | 94 (12.2) |  |  | 342 (87.9) | 47 (12.1) |  |  | | 633 (92.4) | 52 (7.6) |  |  | 1651 (89.5) | 193 (10.5) |  |  |
| Yes | 262 | 98 (80.3) | 24 (19.7) |  |  | 42 (76.4) | 13 (23.6) |  |  | | 67 (78.8) | 18 (21.2) |  |  | 207 (79.0) | 55 (21.0) |  |  |
| Coronary heart disease |  |  |  | 0.097 |  |  |  | 0.632 |  | |  |  | <0.001^*^ |  |  |  | 0.002^*^ |  |
| No | 1810 | 690 (87.5) | 99 (12.5) |  |  | 335 (86.8) | 51 (13.2) |  |  | | 588 (92.6) | 47 (7.4) |  |  | 1613 (89.1) | 197 (10.9) |  |  |
| Yes | 296 | 84 (81.6) | 19 (18.4) |  |  | 49 (84.5) | 9 (15.5) |  |  | | 112 (83.0) | 23 (17.0) |  |  | 245 (82.8) | 51 (17.2) |  |  |
| Chronic obstructive pulmonary disease |  |  |  | <0.001^*^ |  |  |  | 0.025^*b^ |  | |  |  | 0.025^*^ |  |  |  | <0.001^*^ |  |
| No | 1970 | 739 (87.9) | 102 (12.1) |  |  | 365 (87.1) | 52 (12.5) |  |  | | 652 (91.6) | 60 (8.4) |  |  | 1756 (89.1) | 214 (10.9) |  |  |
| Yes | 136 | 35 (68.6) | 16 (31.4) |  |  | 19 (70.4) | 8 (29.6) |  |  | | 48 (82.8) | 10 (17.2) |  |  | 102 (75.0) | 34 (25.0) |  |  |
| Chronic kidney disease |  |  |  | 0.022^*a^ |  |  |  | 0.035^*a^ | |  | |  | 0.009^*a^ |  |  |  | <0.001^*b^ |  |
| No | 2081 | 769 (87.1) | 114 (12.9) |  |  | 381 (87.0) | 57 (13.0) |  |  | | 694 (91.3) | 66 (8.7) |  |  | 1844 (88.6) | 237 (11.4) |  |  |
| Yes | 25 | 5 (55.6) | 4 (44.4) |  |  | 3 (50.0) | 3 (50.0) |  |  | | 6 (60.0) | 4 (40.0) |  |  | 14 (56.0) | 11 (44.0) |  |  |
| Chronic liver disease |  |  |  | 0.182^a^ |  |  |  | >0.999^a^ |  | |  |  | >0.999^a^ |  |  |  | 0.686^b^ |  |
| No | 2093 | 770 (86.9) | 116 (13.1) |  |  | 381 (86.4) | 60 (13.6) |  |  | | 696 (90.9) | 70 (9.1) |  |  | 1847 (88.2) | 246 (11.8) |  |  |
| Yes | 13 | 4 (66.7) | 2 (33.3) |  |  | 3 (100.0) | 0 (0.0) |  |  | | 4 (100.0) | 0 (0.0) |  |  | 11 (84.6) | 2 (15.4) |  |  |
| **Physical examination at admission** | | | | | | | | | | | | | | | | | |  |
| Temperature (℃) |  |  |  | <0.001^*^ |  |  |  | 0.013^*^ |  | |  |  | <0.001^*^ |  |  |  | <0.001^*^ |  |
| <37.3 | 704 | 209 (90.1) | 23 (9.9) |  |  | 112 (93.3) | 8 (6.7) |  |  | | 331 (94.0) | 21 (6.0) |  |  | 652 (92.6) | 52 (7.4) |  |  |
| 37.3-38 | 837 | 320 (89.4) | 38 (10.6) |  |  | 167 (86.5) | 26 (13.5) |  |  | | 262 (91.6) | 24 (8.4) |  |  | 749 (89.5) | 88 (10.5) |  |  |
| 38.1-39 | 514 | 227 (82.8) | 47 (17.2) |  |  | 99 (81.1) | 23 (18.9) |  |  | | 94 (79.7) | 24 (20.3) |  |  | 420 (81.7) | 94 (18.3) |  |  |
| >39 | 51 | 18 (64.3) | 10 (35.7) |  |  | 6 (66.7) | 3 (33.3) |  |  | | 13 (92.9) | 1 (7.1) |  |  | 37 (72.5) | 14 (27.5) |  |  |
| Days from onset to diagnosis (days) | | |  | 0.117 |  |  |  | 0.375 |  | |  |  | 0.067 |  |  |  | 0.003^*^ |  |
| ≤3 | 796 | 196 (87.9) | 27 (12.1) |  |  | 103 (89.6) | 12 (10.4) |  |  | | 425 (92.8) | 33 (7.2) |  |  | 724 (91.0) | 72 (9.0) |  |  |
| 4-7 | 679 | 280 (89.2) | 34 (10.8) |  |  | 117 (83.6) | 23 (16.4) |  |  | | 200 (88.9) | 25 (11.1) |  |  | 597 (87.9) | 82 (12.1) |  |  |
| >7 | 631 | 298 (83.9) | 57 (16.1) |  |  | 164 (86.8) | 25 (13.2) |  |  | | 75 (86.2) | 12 (13.8) |  |  | 537 (85.1) | 94 (14.9) |  |  |
| WBC count (×10^9^/L) | |  |  | 0.001^*^ |  |  |  | 0.147 |  | |  |  | <0.001^*^ |  |  |  | <0.001^*^ |  |
| 4-10 | 1580 | 575 (87.1) | 85 (12.9) |  |  | 289 (85.3) | 50 (14.7) |  |  | | 534 (91.9) | 47 (8.1) |  |  | 1398 (88.5) | 182 (11.5) |  |  |
| <4 | 448 | 179 (89.1) | 22 (10.9) |  |  | 84 (92.3) | 7 (7.7) |  |  | | 143 (91.7) | 13 (8.3) |  |  | 406 (90.6) | 42 (9.4) |  |  |
| >10 | 78 | 20 (64.5) | 11 (35.5) |  |  | 11 (78.6) | 3 (21.4) |  |  | | 23 (69.7) | 10 (30.3) |  |  | 54 (69.2) | 24 (30.8) |  |  |
| Lymphocyte percentage (%) |  |  |  | <0.001^*^ |  |  |  | <0.001^*^ |  | |  |  | <0.001^*^ |  |  |  | <0.001^*^ |  |
| 20-40 | 1190 | 458 (91.4) | 43 (8.6) |  |  | 224 (90.0) | 25 (10.0) |  |  | | 418 (95.0) | 22 (5.0) |  |  | 1100 (92.4) | 90 (7.6) |  |  |
| <20 | 766 | 263 (79.2) | 69 (20.8) |  |  | 129 (78.7) | 35 (21.3) |  |  | | 225 (83.3) | 45 (16.7) |  |  | 617 (80.5) | 149 (19.5) |  |  |
| >40 | 150 | 53 (89.8) | 6 (10.2) |  |  | 31 (100.0) | 0 (0.0) |  |  | | 57 (95.0) | 3 (5.0) |  |  | 141 (94.0) | 9 (6.0) |  |  |
| Lymphocyte count (×10⁹/L) |  |  |  | <0.001^*^ |  |  |  | 0.017^*^ |  | |  |  | 0.057 |  |  |  | <0.001^*^ |  |
| <1 | 1000 | 366 (82.4) | 78 (17.6) |  |  | 173 (82.4) | 37 (17.6) |  |  | | 307 (88.7) | 39 (11.3) |  |  | 846 (84.6) | 154 (15.4) |  |  |
| ≥1 | 1106 | 408 (91.1) | 40 (8.9) |  |  | 211 (90.2) | 23 (9.8) |  |  | | 393 (92.7) | 31 (7.3) |  |  | 1012 (91.5) | 94 (8.5) |  |  |
| Neutrophil percentage (%) |  |  |  | <0.001^*^ |  |  |  | 0.003^*^ |  | |  |  | <0.001^*^ |  |  |  | <0.001^*^ |  |
| 50-70 | 1131 | 433 (90.8) | 44 (9.2) |  |  | 214 (89.9) | 24 (10.1) |  |  | | 393 (94.5) | 23 (5.5) |  |  | 1040 (92.0) | 91 (8.0) |  |  |
| <50 | 246 | 92 (89.3) | 11 (10.7) |  |  | 45 (93.8) | 3 (6.3) |  |  | | 87 (91.6) | 8 (8.4) |  |  | 224 (91.1) | 22 (8.9) |  |  |
| >70 | 729 | 249 (79.8) | 63 (20.2) |  |  | 125 (79.1) | 33 (20.9) |  |  | | 220 (84.9) | 39 (15.1) |  |  | 594 (81.5) | 135 (18.5) |  |  |
| Total | 2106 | 774 (86.8) | 118 (13.2) |  |  | 384 (86.5) | 60 (13.5) |  |  | | 700 (90.9) | 70 (9.1) |  |  | 1858 (88.2) | 248 (11.8) |  |  |

Notes: ^a^ Fisher's exact test; ^b^ Continuity correction; *p value <0.05.
